# A Method for Rapid and Simultaneous Mapping of Genetic Loci and Introgression Sizes in Nematode Species

**DOI:** 10.1371/journal.pone.0043770

**Published:** 2012-08-31

**Authors:** Cheung Yan, Yu Bi, Da Yin, Zhongying Zhao

**Affiliations:** Department of Biology, Faculty of Science, Hong Kong Baptist University, Hong Kong, China; National Cancer Institute, United States of America

## Abstract

*Caenorhabditis briggsae* is emerging as an attractive model organism not only in studying comparative biology against *C. elegans*, but also in developing novel experimentation avenues. In particular, recent identification of a new *Caenorhabditis* species, *C.* sp.9 with which it can mate and produce viable progeny provides an opportunity for studying the genetics of hybrid incompatibilities (HI) between the two. Mapping of a specific HI locus demands repeated backcrossing to get hold of the specific genomic region underlying an observed phenotype. To facilitate mapping of HI loci between *C. briggsae* and *C.* sp.9, an efficient mapping method and a genetic map ideally consisting of dominant markers are required for systematic introgression of genomic fragments between the two species. We developed a fast and cost-effective method for high throughput mapping of dominant loci with resolution up to 1 million bps in *C. briggsae*. The method takes advantage of the introgression between *C. briggsae* and *C.* sp.9 followed by PCR genotyping using *C. briggsae* specific primers. Importantly, the mapping results can not only serve as an effective way for estimating the chromosomal position of a genetic locus in *C. briggsae*, but also provides size information for the introgression fragment in an otherwise *C.* sp.9 background. In addition, it also helps generate introgression line as a side-product that is invaluable for the subsequent mapping of HI loci. The method will greatly facilitate the construction of a genetic map consisting of dominant markers and pave the way for systematic isolation of HI loci between *C. briggsae* and *C.* sp.9 which has so far not been attempted between nematode species. The method is designed for mapping of a dominant allele, but can be easily adapted for mapping of any other type of alleles in any other species if introgression between a sister species pair is feasible.

## Introduction

*C. briggsae*, a close relative of *C. elegans* has gained increasing attention in biomedical research in recent years. As a companion species, it has been frequently used for studies of comparative genomics [1]–[4], genome evolution [1], [5], gene prediction improvement in *C. elegans* [6], identification of regulatory elements [7], [8], population genetics [9], [10] as well as developmental dynamics of various developmental pathways [11], [12]. Recent effort in sampling new nematode species has identified multiple novel *Caenorhabditis* species [13]. Most of these new species are more related to *C. briggsae* than to *C. elegans* in the phylogenetic analysis. One of these species, *C.* sp.9 is able to mate and produce hybrid viable progeny with *C. briggsae* [14], hereafter termed as its sister species, providing an unprecedented opportunity for studying the genetic and molecular mechanisms of hybrid incompatibility (HI) between the nematode species. Unfortunately, such a sister species is still lacking for *C. elegans*, a well-established model organism, preventing its use in such study. Despite the first ever genes responsible for intra-species HI have recently been identified in *C. elegans* using SNP based mapping [15], nematode species have never been used for isolation of any other loci or genes underlying inter-species HI phenotypes due to the following reasons. First, lack of a sister species with which *C. elegans* can mate and produce viable progeny [16] prevents its use in identification of HI loci or genes between other species. Second, mapping of HI loci between related species frequently involves an introgression process, in which a labeled genomic fragment from one species is introduced into the genetic background of the other by repeated backcrossing. If the genomic fragment in an otherwise genetic background of its sister species produces compromised fitness, the fragment is held responsible for the HI. The key to the HI mapping is to develop chromosomal markers evenly distributed over the genome of the parent species. Ideally the markers are dominant and visible ones so that crossing progeny carrying the marker and its associated genomic fragment can be readily identified for the next round of crossing in the heterozygous hybrid progeny. However, lack of dominant and visible genetic markers over the genomes of any nematode species as well as its effective mapping method with high resolution inhibits the study of HI loci in the nematode species. In *Drosophila* species, flagging and mapping of a chromosomal fragment are primarily achieved by a combination of P element transposes with a dominant and visible *white* gene as a marker [17]. The former allows the random insertion of a transgene into a *Drosophila* genome while the latter permits the selection of the transgenic animals from those without transgene insertion.

To facilitate the HI study using the species pair of *C. briggsae* and *C.* sp.9, a genetic map consisting of a dominant and visible maker in *C. briggsae* would be invaluable for nailing down a specific genomic region producing HI phenotypes. Generation of such map faces two major challenges. First, evenly flagging of *C. briggsae* chromosomes with a dominant and visible maker; second, mapping of the makers into a defined genomic region. In terms of the first challenge, few existing mutants demonstrate dominant and easily identifiable expression, thus a new type of marker has to be developed. As to the second challenge, there are a few methods established for genetic mapping in *C. briggsae* [18], [19]. However, these methods were all based on bulked segregant analysis (BSA), one especially designed for mapping of a recessive allele but not convenient for mapping of a dominant one. In addition, the methods suffer from either low resolution [18] or high costs [19]. For example, we have previously developed an SNP-based oligonucleotide array for genetic mapping in *C. briggsae* based on the BSA [19]. The method works well for the recessive mutations with relatively high mapping resolution, but involves substantial costs for manufacturing the customized microarray. It also demands sophisticated instrumentations for hybridization and signal scanning that may be beyond of reach for many small labs. Thus an efficient and cost-effective method for mapping of a dominant allele is necessary in *C. briggsae*. To this end, we developed a new method and tested its use by mapping of C. *briggsae* dominant transgenic markers.

## Materials and Methods

### Strains and maintenance

*C. briggsae* AF16 (sequenced reference strain), HK104, *C.* sp.9 JU1421 (inbred derivative of JU1325, a gift from Asher Cutter), *C.* sp.9 JU1422 (inbred derivative of JU1325, a gift from Marie-Anne Félix), *C.* sp.9 EG5268 (a wild isolate from Congo, a gift from Asher Cutter), *C.* sp.9 ZZY0050 (a derivative of EG5268 after 25 generation of inbreeding), RW20000 (*cbr-unc-119(st20000*), III), ZZY0021 (*cbr-unc-119*, *zzyIs0021*[cbr-myo-2p::GFP, *unc-119*(+)], II), RW20101 (*cbr-unc-119*, *stIs20101*[cbr-myo-2p::GFP, *unc-119*(+)], X), RW20105 (*cbr-unc-119*, *stIs20105*[cbr-myo-2p::GFP, *unc-119*(+)], IV), RW20120 (*cbr-unc-119*, *stIs20120*[cbr-myo-2p::GFP, *unc-119*(+)], X), ZZY0013(*cbr-unc-119*, *zzyIs0013*[cbr-myo-2p::GFP, *unc-119*(+)], I), ZZY0015(*cbr-unc-119*, *zzyIs0015*[cbr-myo-2p::GFP, *unc-119*(+)], III), DY250 (*cby-pry-1(sy5353)*, I), PS9148(*cbr-sma-6(sy5148*), II), PS9357 (*cbr-unc-4(sy5341*),II), PS9022(*cbr-dpy(sy5022*),III), BC1972(*cbr-unc-22?(s1270*), IV), BC6031(*cbr-unc(sy5094*),V), PS9454(*cbr-unc(sy5415*),V), PS9001(*rot-1(sy5001*), X). All the worm strains were maintained on NGM plate with 1.5% agar seeded with OP50 *E. coli* and kept in a 25°C incubator unless during setting up of the crossings at room temperature.

### Generation of stable transgenic *C. briggsae* strains expressing GFP

A reporter construct (pZZ0031) consisting of a pharyngeal specific promoter from *cbr-myo-2* fused with GFP was built for generation of stable transgenic strains in *C. briggsae*. It also carries a *C. elegans unc-119* rescuing fragment as a selection marker for bombardment. The reporter construct was randomly integrated into the *C. briggsae* genome by bombarding *cbr-unc-119* mutant we isolated previously as described [20]. Independent transgenic lines showing 100% rescue and expressing bright GFP in the pharynx under a stereo fluorescent microscope were used in the mapping.

### Introgression between the GFP labeled *C. briggsae* strains and *C.* sp.9

**Figure 1:**
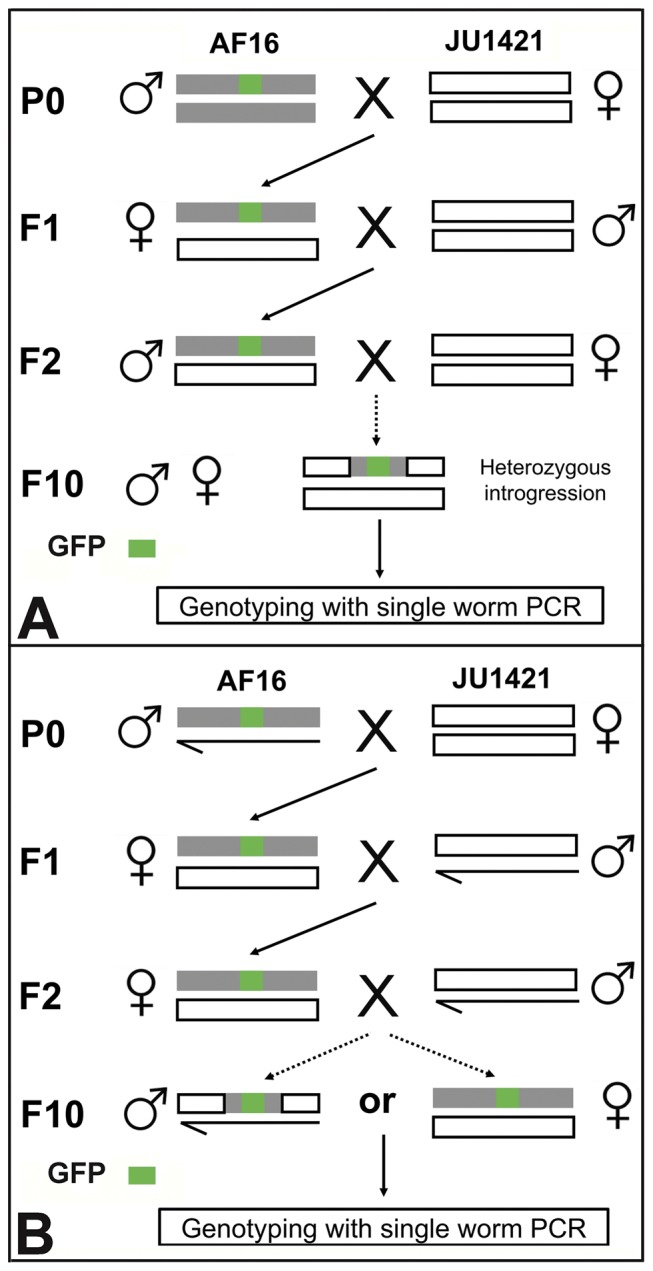
Introgression strategies between *C. briggsae* and *C.* sp.9. A. Introgression for the autosome linked marker. B. Introgression for the X chromosome linked marker. See text for more details.

The GFP linked *C. briggsae* genomic fragments were introduced into an otherwise *C.* sp.9 genetic background by repeated backcrossing, a process called introgression (Figure 1). The introgression basically followed the scheme used in *Drosophila* [21], [22] with modifications. Five young adult or L4 males of the GFP labeled *C. briggsae* animals were mated with seven L4 females of *C.* sp.9 strain JU4121 (Figure 1) or EG5268 (data not shown). Given the fact that most F1 hybrid male progeny between *C. briggsae* and *C.* sp.9 were inviable and/or sterile [14], five GFP positive L4 female (obligated female rather than hermaphrodite here) of F1 hybrid progeny were mated with seven L4 or young adult JU1421 or EG5268 males on individual plates in two replicates. For autosome linked GFP strains, seven F2 males were crossed with five JU1421 or EG5268 L4 females. For X-linked GFP strains, there are two scenarios. First, if the ratio between GFP positive and GFP negative males is roughly 1∶1 in the subsequent hybrid progeny, crossings with JU1421/EG5268 were performed by alternating GFP positive males and GFP positive females due to the X chromosome linkage. Second, if the large X effect was observed, i.e., GFP positive males were rarely observed in the subsequent hybrid progeny, only the GFP positive L4 females were picked for crossing with JU1421 or EG5268 males. The crossings were repeated up to 15 generations.

### Mapping of the GFP integration loci in *C. briggsae* and introgression sizes in *C.*sp.9 by PCR

**Figure 2:**
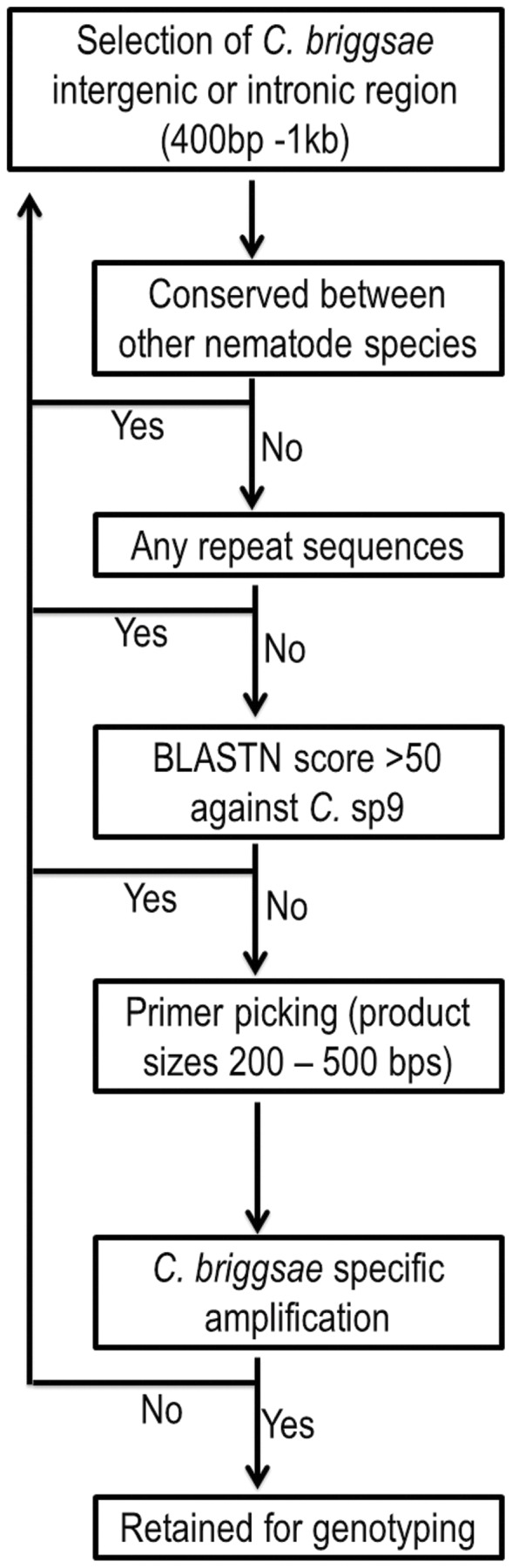
Flow chart for the selection of *C. briggsae* specific primers. See text for more details.

The introgression serves as three purposes, mapping of GFP integration positions in *C. briggsae* genome, generation of introgression lines and estimation of the introgression sizes. On average roughly15 pairs of PCR primers specific for *C. briggsae* genomic sequence were selected for each chromosome with roughly equal distances from one another except for those located in the middle of the chromosome (See Table S1). The primer density was slightly lower in the middle of the chromosome than those in the left and the right arms where the increased recombination rate was found [1]. To maximize the chance of the specific amplification of genomic fragment from *C. briggsae* but not from *C.* sp.9, a fragment of interest was manually chosen from either the intronic or the intergenic region of *C. briggsae* genome with the least conservation as opposed to its orthologous region of *C. elegans*. Also excluded were those located within the repeated regions highlighted by the repeat masker [23]. The chosen sequences were aligned against *C.* sp.9 genome with BLASTN using default settings except with the repeat masker filter “off” on the 959-nematode server (Blaxter, personal communication). Only those regions containing no hits with a BLAST score of more than 50 were retained as a target for PCR primer selection using Primer 3 [24]. If the sequence did not meet the criteria, a nearby sequence was selected as an alternative until the above criteria were met (Figure 2). The selected primers were used as a query to align against *C.* sp.9 genome using the same BLASTN as described above. Those with more than eight base pair matching exactly the *C.* sp.9 genome sequence at the 3′ends were discarded and the primer selection process was reiterated until the primers that satisfied the above constraint was found nearby. A total of 94 pairs of PCR primers were picked (See Table S1). Seven of them still amplified a band with expected sizes from both *C. briggsae* (AF16) and *C.* sp.9 genomic DNA (ZZY0050) and were discarded (Data not shown).

The mapping was started by genotyping of either autosome or X chromosome by three pairs of the primers one each from left, middle and right arm using the single worm PCR with GFP positive post-introgression worm as a template. A single GFP positive adult was lysed in the 0.5 ul lysis buffer with proteinase K with the following incubation steps: −80°C for one hour, 65°C for 90 minutes followed by 95°C 15 minutes to inactivate the proteinase K. The PCR were performed in a 20 ul volume with Applied Biosystems® AmpliTaq Gold® Fast PCR Master Mix with the following conditions: 95°C 10 min for 1×; 96°C 3 seconds, 55°C 3 seconds and 68°C 10 seconds for 30 cycles; 72°C 10 seconds for 1 cycle. The linkage of the GFP locus with autosome or X chromosome was readily determined during the introgression process. Once it was anchored to the arm of a specific chromosome, other primers located within the same chromosomal arm were used for PCR amplification to narrow down the GFP and its linked region.

### Mapping through visible marker

Two-point linkage mapping of the GFP locus in *C. briggsae* was performed using various visible but recessive mutations available for different chromosomes (Table S2). Five GFP positive males were mated with seven young adults for each mutant in two replicates. Five F1 wild type GFP hermaphrodite adult animals were picked onto a single plate and allowed for selfing. Linkage of the GFP with the mutations was scored in the F2 populations by examining the ratio between GFP positive and negative mutant animals. A total of eight *C. briggsae* visible mutants were used in the mapping. A single visible mutant was used for each linkage group except for chromosome III and V, for which two different mutants were used. The genetic and/or physical positions as well as the phenotypic data of each mutant were listed in Table S2.

### Mapping through inverse PCR

Given the relatively big size of the construct (12756 bps), a total of four pairs of inverse PCR primers were selected for mapping of the potential breaking points of the GFP reporter construct pZZ0031 in the *C. briggsae* genome (Figure S1). The primers spanned the four boundaries between the GFP coding and *C. elegans unc-119* rescuing sequences. This is based on the assumption that both the GFP and ce-unc-119 (+) rescuing sequences were intact in the transgenic animals expressing GFP. The primer positions and its combination with corresponding restriction digestion plans were listed in the Figure S1.

### Mapping through SNP-based Comparative Genomic Hybridization (CGH) array

The mapping was done using a customized oligo array as described previously [19] except that the number of F2 homozygous GFP animals was not 100 but 5–10 (data not shown).

### Estimation of the introgression size

This was done in the same way as that used for mapping of the GFP integration site in *C. briggsae*. Roughly 15 pairs of primers were selected for each chromosome. Additional primers were selected for further narrowing down positions of the introgression fragment. The sizes of the introgression fragment were estimated to a range based on the absence and presence of a PCR product by the primers with defined genomic coordinates.

### Gene prediction of *abce-1* in *C. sp.9* and phylogenetic analysis

**Figure 3:**
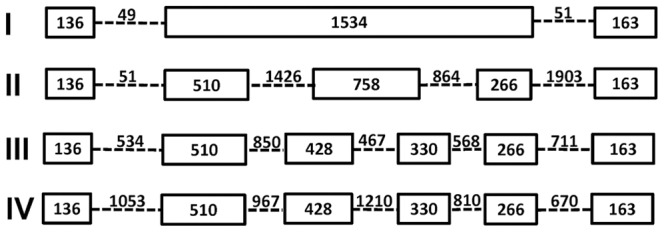
Intronic structure of *abce-1* genes in nematode species. I: *C.* sp.9; II: *C. elegans*; III: *C. briggsae*; IV: *C. brenneri*. Exons are denoted as hollow bars with its size in bp labeled inside while introns (not in scale) shown as dashed lines with sizes labeled above.

*C. briggsae* ABCE-1 protein sequence was retrieved from Wormbase (WS230) and used as a query to search for its homologous genomic region in *C.* sp.9 using TBLASTN with default parameters in the 959 nematode genome server (Blaxter, personal communication). The sequence for the hit with the highest score was downloaded and used as an input for *ab initial* gene prediction program FGENESH [25] with default parameters specific for *C. elegans*. The predicted gene structure (intron/exon boundaries) was manually verified and compared to that of its orthologues in *C. elegans* and *C. briggsae* (Figure 3). The predicted ABCE-1 protein sequence along with those of *C. briggsae, C. brenneri* and *C. elegans* were used as input for multiple sequence alignment with ClustalX [26]. The aligned sequences were used for constructing a Neighbor Joining (NJ) phylogenetic tree with the default settings. The tree was subject to bootstrapping for 1000 times (Figure S2).

### Establishing of inbreeding line of EG5268

Five L4 males were crossed with five L4 females on a regular NGM (1.5% agar) plate at 25°C. The crossings were repeated using their progeny for a total of 25 generations in two replicates to give rise to two independent inbreeding lines, ZZY0050 and ZZY0051.

### Microscopy

GFP positive worms were screened under a Leica M60 fluorescence stereomicroscope according to the manufacturer's description.

## Results and Discussion

### Overall mapping strategy

**Figure 4:**
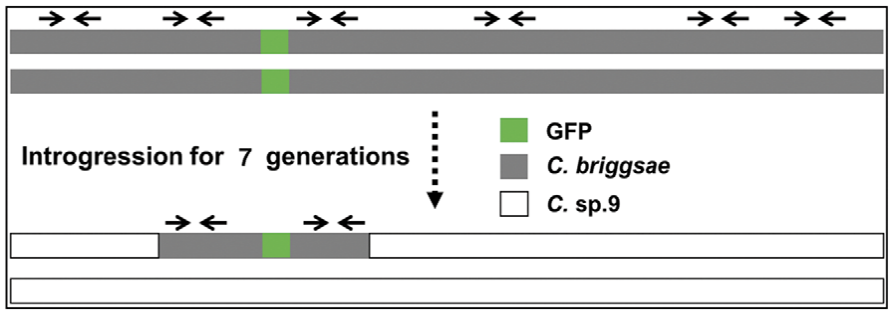
Strategy for mapping of a dominant GFP (green bar) marker in *C. briggsae*. *C. briggsae* specific primers (paired arrows) across a chromosome were selected that would specifically amplify the fragment of interest in *C. briggsae* genome (grey bar) but not that from its homologous region in *C.* sp.9 (white bar). After multiple generations of backcrossing (introgression) into *C.* sp.9 background using the GFP positive hybrid progeny, only the *C. briggsae* genomic fragment that is closely linked to the GFP locus will be retained in the hybrid progeny due to the recombination. The sizes of the GFP linked *C. briggsae* fragment can be judged by the single worm PCR using a hybrid animal either heterozygous or homozygous (not shown) for the GFP locus as a template. Presence or absence of PCR product will allow simultaneous estimation of the approximate location for the GFP insertion in the *C. briggsae* genome as well as the calculation of the introgression size in the *C.* sp.9 background.

To facilitate rapid mapping of dominant loci in *C. briggsae*, we developed a straightforward mapping method that took advantage of introgression between *C. briggsae* and its sister species *C.* sp.9 followed by genotyping with single worm PCR using *C. briggsae* specific primers (Figure 4). Special considerations for the method include the scalability and efficiency for mapping of a dominant allele, which would be inconvenient using any mapping methods that were based on the bulked segregant analysis. Given the fact that a substantial portion of the hybrid progeny between *C. briggsae* and *C.*sp.9 are viable [14], we reasoned that if we cross a *C. briggsae* dominant marker into *C.* sp.9 and then repeatedly backcross (introgression) the hybrid progeny expressing the marker with *C.* sp.9 for multiple generations, only a minimal amount of *C. briggsae* genomic fragment that is closely linked to the marker will be retained in an otherwise *C.* sp.9 background due to recombination. We would then be able to estimate the sizes of the introgression fragment can by single worm PCR with *C. briggsae* specific primers (Figure 4). The presence or absence of a PCR product indicated *C. briggsae* or *C.* sp.9 specific regions respectively (Figure 4), providing an estimate of not only the marker's genomic position in *C. briggsae*, but also the size of the introgression fragment in the *C.* sp.9 background, which will be invaluable for mapping of the loci underlying an observed HI phenotype between the two species.

As a proof of principle, we set out to map chromosomally integrated transgenic GFP loci, which serve as a dominant and visible marker for the introgression (see below). We successfully mapped the locus into a region as small as 0.9 million bps with a few crossings (Table 1). We have so far used the method for mapping of over 60 independent transgenic strains stably expressing GFP or mCherry (manuscript in preparation). As with other recombination-based methods, the mapping resolution is dictated by the recombination frequency during meiosis. The resolution for both mapping and introgression breaking points can be further improved by increasing the density of the PCR primers and the number of generations for backcrossing. The mapping resolution seems higher for those located within the autosomal arms but lower for those located within middle of autosome and X chromosome which is in agreement with previous observations [1] (Table 1). It is worthy of noting that the mapping process will also contribute to the production of introgression lines which will be the essential reagents for mapping of a HI locus (Figure 4).

**Table 1:** Summary of the mapping results.

Strain name	Linkage group	Physical position	Introgression size
ZZY0013	Chrom I	2.1 to 3.0 Mb	0.9 Mb
ZZY0021	Chrom II	6.1 to 11.1 Mb	4 Mb
ZZY0015	Chrom III	7.1 to 9.6 Mb	2.5 Mb
RW20120	Chrom X	1.5 to 6.0 Mb	4.5 Mb

### Selection of *C. briggsae* specific primers

The key to the successful mapping is selection of *C. briggsae* specific primers. Given the close relationship between the two species [13], stringent measures must be taken to ensure the specific amplification of the genomic sequences of *C. briggsae* but not that from *C.* sp.9 (Figure 2). We attempted to target those regions that are located either inside an intron or an intergenic region to avoid potential conservations more likely to be seen between the coding sequences of nematode species. Since the genome sequence of *C.* sp.9 is in its infancy, i.e, only a collection of relatively short contigs are available for alignment (http://www.nematodes.org/), we took advantage of comparative genomics tools provided in UCSC genome browser [27] to prioritize the *C. briggsae* genomic regions for primer selection (See Materials and Methods). We selected a total of roughly 15 pairs of primers on average for each individual chromosome. They are evenly distributed from the left to the right arm but with a slightly higher density in the arms than that in the middle of a chromosome. Additional primers can be picked whenever needed in between the existing primers for narrowing down the location of a specific introgression fragment. Approximately 10% of the selected primers gave rise to the identical PCR product with the genomic DNAs from both species as a template (data not shown). We reiterated the primer selection process until a satisfactory primer pair was achieved (Figure 2). A total of 87 pairs of the primers were selected for the *C. briggsae* genome (cb3 assembly). The average distances between adjacent primer pairs are roughly 1.1 M bps. A complete list of the primer sequences and its genomic coordinates (WS230) can be found in the Table S1.

One complication is that *C.* sp.9 genomic sequence seems to carry contamination from that of *C.* sp.7 during its genomic sequencing (Patrick Mink, personal communication). Consistent with this, we found that gene *abce-1* ortholog in *C.* sp.9 does not cluster with its equivalent in *C. briggsae* but looks like a outgroup member in both intronic (Figure 3) and phylogenetic analyses (See Figure S2). *abce-1* of *C. sp.9* contains a total of three exons while that of other three species, *C. elegans,C. briggsae* and *C. brenneri* carries a total of six exons though the sizes of the coding sequences remain the same among the four species (note, a mis-prediction of *C. briggsae abce-1* gene was manually corrected by removal of six bps in its fourth exon, leading to the size changed from 336 (Wormbase WS230) to 330). *C.* sp.9 *abce-1's* second exon could represent an ancestral version while its equivalent in other nematode species is likely subject to two events of intron gain, resulting in split exons. ABCE-1 is a well-conserved protein encoded by a single copy gene in the genomes of most eukaryotic species. It was annotated as an RNAse L inhibitor, but was also involved in both transcription and translation [28]. Given its extreme conservation and lack of redundancy, ABCE-1 protein sequences were used for inference of phylogenetic relationship in nematode species [2]. Based on the established phylogenetic relationships [13], we expect the intronic structure of *C.* sp.9 would be more similar to that of *C. briggsae* or *C. brenneri* than to that of *C. elegans*. However, the unexpected intronic structure and phylogenetic relationship as demonstrated the phylogenetic tree (Figure S2) suggest that this part of the genomic sequence might be derived from *C.* sp.7 which seems an outgroup member for elegans group [13]. We were not being able to estimate to what extent the released *C.* sp.9 genomic DNA was contaminated with that of *C.* sp.7. Such contamination could be responsible for some of our failed primers in genotyping. Thus a more reliable version of the *C.* sp.9 genome sequence is necessary for efficient mapping and cloning of loci underlying an observed HI phenotype between the two species.

Another limitation for the method is that there are many random genomic sequences that were assigned onto a chromosome but their chromosomal coordinates remain unknown in *C. briggsae* [6]. A few of our selected primers can produce *in silico* identical PCR product in both the chromosomal regions and those unassigned regions (Table S1). We were not certain whether these represent assemble errors or the real duplicated genomic regions. In addition, roughly 7 M bps genomic sequences were not anchored onto any chromosome [1]. Thus we were not able to select any primers therein for our mapping purpose and could possibly skew the even distributions of our selected primers over the genome. Further work is needed to improve the *C. briggsae* genome assembly.

### Introgression strategies

Given the low fertility of the F1 hybrid males [14] and potential linkage of the GFP locus with either autosome or X chromosome, we adopted two separate introgression strategies for the autosome or X chromosome linked GFP loci (Figure 1). In both cases, GFP labeled *C. briggsae* young adult males in an AF16 background were initially mated with *C.* sp.9 (JU1421) L4 females and the GFP positive F1 female progeny were mated again with JU1421 L4 males. For introgression with autosome linked GFP, the GFP positive F2 hybrid males were mated with the JU1421 L4 females and repeated the crossing in the same direction from F2 up to 15 generations (Figure 1 & 4). For introgression with X chromosome linked GFP, we performed the crossing using the following two strategies. If the large X-effort was observed, only GFP positive females were used for the subsequent crossings. If the large X-effort was not observed, the GFP positive F2 L4 males were used for mating with JU1421 females. The subsequent crossings would alternate with opposite crossing directions using GFP males and GFP females due to the linkage (data not shown). Crossings were performed at least for seven generations before genotyping with PCR. A single male or female that expressed GFP was used as a template in single worm PCR reaction. Unlike the mapping based on the bulked segregant analysis, it is unnecessary to render GFP locus homozygous before the PCR, making it especially convenient for mapping of a dominant locus.

### PCR genotyping

**Figure 5:**
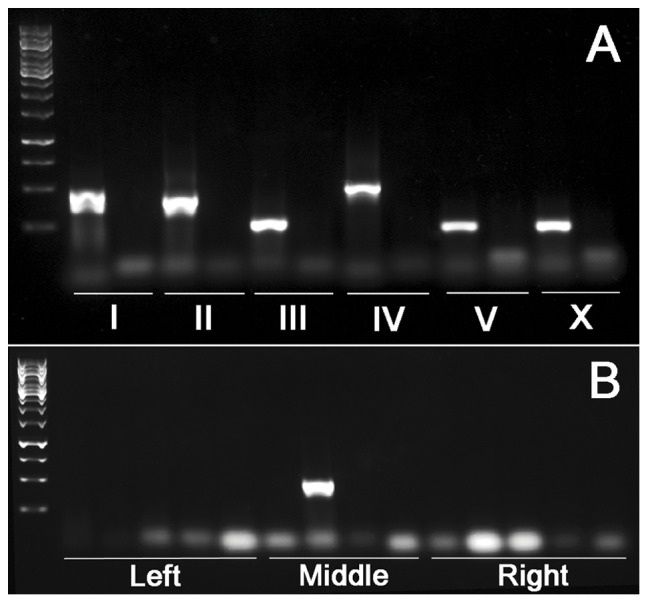
PCR genotyping results. A. Test of the primer specificities. A side-by-side comparison of the agarose gel pictures of the PCR products with the genomic DNAs of AF16 (left) or JU1421 (right) as a template. Only a single PCR product was shown for each chromosome with their identities indicated in the bottom. B. Mapping of a GFP locus onto the middle of chromosome II using the GFP positive hybrid progeny (after 15 generations of introgression) as a template. A total of 14 pairs of primers (ordered from left to right based on their genomic coordinates) were used and only a single pair of primers gave rise to PCR product with expected size, indicating the GFP insertion site is located between the boundaries defined by its two adjacent pairs of primers. Chromosomal arms are defined into “Left”, “Middle” and “Right” based on their relative genomic coordinates.

To test the specificities of the selected primers, we performed PCR reactions with purified genomic DNAs from both *C. briggsae* (AF16) and *C*. sp.9 (JU1421 or EG5268) as a template. We only retained those primers in the subsequent genotyping assay that produced a single PCR product with expected size using *C. briggsae* genomic DNA as a template but yielded no band with *C.* sp.9 genomic DNA as a template (Figure 5, Figure S1). For those that produced identical PCR bands with DNA templates from both species or yielded multiple bands with the DNA template of *C. briggsae*, the primers were discarded and an alternative one within the adjacent location was selected and tested for its specificity. The selection process was reiterated until the above criteria were met for all the primers. We then used them for genotyping by performing single worm PCRs with a single AF16 or JU1421/EG5268 adult as a template using the same PCR conditions as those with genomic DNA. We found the PCR results using a single worm as a template agree well with those using genomic DNA (data not shown).

To minimize the number of PCR reactions in the initial mapping, instead of running 74 or 13 single worm PCR reactions for autosome linked or X-linked GFP locus respectively (Figure S1), we chose to perform 15 PCR reactions with three pairs of primers from each autosome representing its left, middle and right arm respectively or three PCR reactions for X chromosome. By doing so, we found it is very likely to anchor a GFP locus onto an arm of a specific chromosome. Further narrowing down the introgression breaking points can be achieved by performing the PCR using complete set of the primers specific for the chromosome (Figure 5). Thus, a mapping resolution of 1–2 M bps can be readily achieved with approximate 30 or 13 PCR reactions for the autosome or X linked marker respectively, which is much higher than that from other mapping method (Table 1) [18]. The mapping method eliminated the requirement of rendering homozygous the target locus as is the case for those based on bulked segregant analysis. The mapping resolution is primarily dictated by the recombination frequency, thus increasing the number of generations for crossing will in theory improve the mapping resolution. However, we found that after 15 generations of introgression, the mapping resolution seems not benefit much by further increasing the number of crossing (data not shown). Usually introgression of 7 to 8 generations is sufficient for mapping of a locus into genomic region ranging from approximately 1 to 5 M bps chromosomal region depending on the chromosomal positions of GFP transgene.

### Comparisons of mapping using other methods

We attempted a few other *C. briggsae* mapping methods before we landed with the current one through introgression followed by PCR genotyping. First, we tried two-point mapping with a handful of available genetic mutants (See Table S2). We were able to assign a few transgenes onto a specific chromosome correctly for most of the mapped strains (data not shown). However, we found the mapping efficiency was relatively low in term of resolution and labor costs. Since we did not have enough visible markers across the different parts of the *C. briggsae* genome (Table S2), we could not improve the mapping resolution further with the method. Sometimes, we found that some of the transgenes were assigned onto an incorrect chromosome presumably due to a large distance between the marker and the GFP locus. On the other hand, many mutants were either unhealthy or demonstrate slow growth (Gupta, personal communication), leading to the skewed phenotypic scorings. In addition, some mutant phenotypes were not readily recognizable especially by a novice. Thus, we reason that time spent with the method may not be warranted because further mapping is still needed to improve resolution.

Secondly, we attempted the method of inverse PCR in a hope to definitely locate a transgene insertion site for three independent strains. Given the rescued wild type and GFP expressing worms were used in the genotyping, we assumed both the GFP coding and the *C. elegans unc-119* rescuing sequences were intact and four pairs of the inverse PCR primers were picked covering the sequences in between the two intact fragments (Figure S1). We performed a total of 18 PCRs for three different transgenic GFP positive strains (two pairs of the primers formed four different combinations with alternative restriction plans). In contrast to our expectations, all the PCR products were unambiguously derived from the vector itself based on both PCR product sizes and sequencing results of the PCR products (data not shown), suggesting that in few cases the transgene was inserted as a single copy but likely as multiple copies albert at a low copy number.

We then tested the mapping using the SNP-based oligonucleotide microarray we had developed previously [19]. The mapping results agreed well with those using the current method (data not shown). However, it involved substantial costs mostly due to manufacturing of a customized chip plus the subsequent hybridization costs (data not shown). In addition, the instrumentations for the microarray might also be beyond of reach by many small labs because it was customized for NimbleGen microarray platform.

Compared to the above methods, the introgression based mapping is relatively more feasible and cost effective and especially convenient for mapping of a dominant allele. The method was designed for mapping of a dominant locus in *C. briggsae* but would be applicable to any other nematode species if a sister species pair is available. It can be easily adapted for mapping of a recessive allele. The current mapping method is not without limitations. One of the major disadvantages is the decreasing efficiency in genetic map construction once the markers reach certain density over the genome. Specifically, we started the mapping blindly in terms of the allele locations. Thus, once many independent markers were positioned at different locations over the host genome, it is more likely that the strain newly chosen for mapping may lie in the close proximity to a mapped one, leading to redundant mapping/introgression of the similar or same genomic fragment. This could be partially alleviated using a multiplex SNP-based microarray. We have attempted the mapping using a 12× chip that achieved a mapping resolution up to approximately 2 Mb with much reduced costs compared to 1× chip (data not shown).

### Possible interbreeding between *C.* sp.9 and *C. briggsae* in the wild

One of the major motivations for this work is to help develop a *C. briggsae* genetic map that consists of dominant and visible markers with resolution up to 1 M bps. The map will facilitate systematic identification of specific loci in *C. briggsae* that produce hybrid incompatibilities in an otherwise *C.* sp.9 background. Our mapping protocol relied on the introgression between *C. briggsae* and *C.* sp.9, thus the genetic background of both species would be critical for the successful mapping. This is more relevant to *C.* sp.9 than to *C. briggsae* due to the following reasons. First, *C. briggsae* starting strains for introgression were all derivatives of AF16, the genetically homogenous strain used for genome sequencing [6]. Second, our targeted genomic regions used for primer selection were biased for the sequences with fast divergence rate and thus its equivalent regions in *C.* sp.9 may have a higher chance to be eliminated in different wild isolates. Third, the gonochoristic mode of reproduction makes it more likely heterogeneous genetically. Fourth, given the substantial viable hybrid progeny produced between *C.* sp.9 and *C. briggsae*, strains isolated in different locations might have different level of introgression between the two species if they happened to share the habitats which might not uncommon based on their known habitats [13].

**Figure 6:**
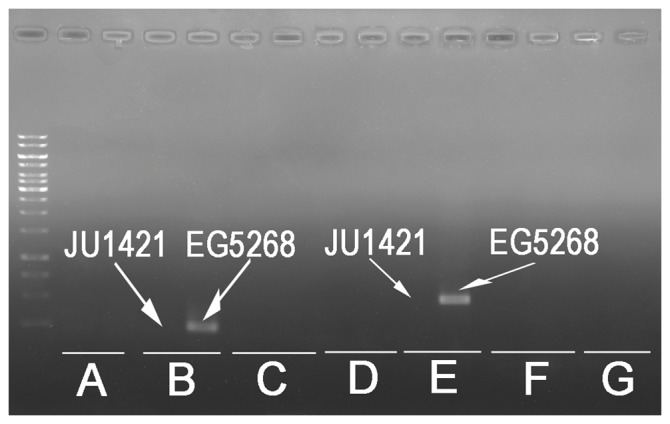
Amplification of the PCR products with the sizes expected for AF16 using the genomic DNAs of EG5268 but not that of JU1421 as a template for two out the 7 pairs of primers. A side-by-side comparison of PCR amplification using the genomic DNAs of JU1421 (left of each pair) or EG5268 (right of each pair) with the primers specific for the chromosome I. Two unexpected amplifications with EG5268 were shown. Only results of seven pairs of primers (A to G) were shown. All of the primers gave rise to the expected PCR amplifications with AF16 genomic DNA as a template (data not shown).

We had attempted introgression using another strain of *C.* sp.9, EG5268, a wild isolate from Congo Republic, which produced a higher number of progeny than that of JU1421 [29]. Roughly one tenth of our primers gave rise to a positive PCR band with the same size as that from *C. briggsae* when EG5268 genomic DNA was used as a template (Figure 6, Table S1). It is plausible that substantial introgression could take place between *C.* sp.9 and *C. briggsae* in the wild habitats based on their known ecology [14]. However, levels of the introgression between the two are likely to be strain dependent, i.e., JU1421 and EG5268 may have independent introgression with *C. briggsae*. It could also be possible that fast divergence of the targeted genomic regions for the primer selection that underlie the differential amplification in both strains. The EG5268 strain was subject to 25 generations of inbreeding crossings before being used in our introgression. Since introgression progeny between the AF16 derived strains and EG5268 seems healthier than that between AF16 and JU1421 (data not shown), different HI loci are likely to be isolated when EG5268 was used for introgression. Thus genome sequencing of an inbreeding line of EG5268 is likely to provide a better framework for HI research using the species pair.

### Potential errors in *C. briggsae* genome assembly revealed by the mapping results

**Figure 7:**
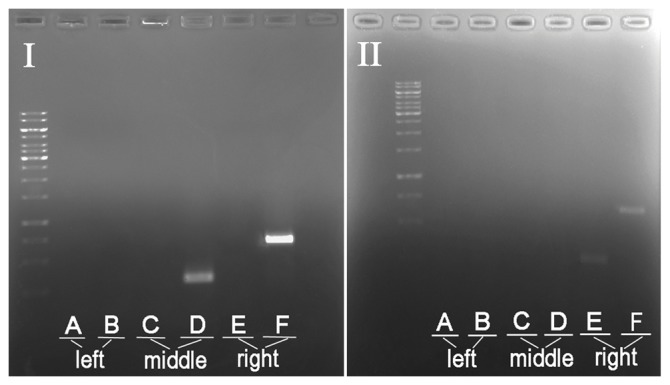
PCR amplification of discontinuous genomic fragments on the same chromosome using animals from one introgression lines as a template. Shown were the PCR amplifications with six pairs of primers (A–F) specific for the chromosome X using a GFP positive adult worm from two different introgression lines as a template after 15 generation of introgression. The PCR product was ordered by the genomic coordinates of the selected primers from the left to the right arm. Note the amplifications of the discontinuous regions as indicated by the primers D and F but not by E (panel I) as opposed to the amplifications of a continuous region as indicated by the primers E and F but not the primer D (panel II).

To our surprise, we observed some of the GFP loci were simultaneously anchored on the different regions of the same chromosome (Figure 7) or even on the different chromosomes (data shown). We speculate that one of the possible reasons is likely due to the assembly errors of the *C. briggsae* genomic contigs of the same or between different chromosomes. The genomic regions targeted for the primer selection were more prone to the possible assembling errors due to requirement of maximum sequence divergence. It could also be possible that some cryptic genetic modifiers contribute to the co-segregation of the observed genomic fragments during the introgression. Further validations need to be done to verify the possibility. Recent re-annotation of *C. briggsae* genome using the RNA-seq data of messenger RNA significantly improves the accuracy of the predicted gene set in the species [30]. A re-assembly of *C. briggsae* genome using the genotyping data of recombinant inbred lines help anchor majority of the unanchored contigs on to chromosome [31] but it would be invaluable to fill the *C. briggsae* genomic gaps and anchor the remaining unassigned or random sequence contigs [1] onto their proper genomic contexts.

## Conclusions

Hybrid incompatibility plays an essential role in generation of biodiversity. Isolation of genetic loci underlying HI phenotypes demands a pair of closely related sister species in addition to various genetic and molecular tools. Thus, model organisms were frequently used for studying the genetic and molecular mechanisms of HI between a pair of sister species [32]–[35]. Unfortunately, *C. elegans* as a model organism contributes little in the field due to lack of the sister species with which it can mate and produce viable progeny. Recent identification of the sister species *C.* sp.9 of *C. briggsae* opens the possibility to use the species pair in isolation of HI loci between nematode species. However, lack of genetic markers and efficient mapping method inhibits its use in HI study. Here we developed a rapid mapping method with high resolution for mapping of dominant loci in *C. briggsae*, which is invaluable for isolation of HI loci between the two nematode species. We have used the method to successfully map over 60 stable transgenic lines expressing various markers (manuscript in preparation) into defined genomic regions as small as 900 kilo bps. The method was designed for mapping of a dominant locus but can be readily adapted for mapping of any other loci. The mapping method will greatly facilitate construction of a genetic map consisting of dominant markers and pave the way for systematic isolation of HI loci between *C. briggsae* and *C.* sp.9 which has so far not been attempted between nematode species.

## Supporting Information

Figure S1 Relative positions of PCR primers and its combinations with restriction enzymes as shown in the context of the restriction map of the construct pZZ31 that was used to bombard the *cbr-unc-119* mutant for generation of the stable transgenic line expressing GFP. The ORFs are self-explanatory.
(TIF)

Figure S2 The unrooted Neighbor-Joining (NJ) tree constructed with multiple alignment using ABCE-1 protein sequence from *C. elegans* (ABCE-1), *C.* sp.9(Csp9-ABCE-1), *C. briggsae* (CBR-ABCE-1) and *C. brenneri*(CRE-ABCE-1). The number of bootstrap support from 1000 replicates was shown.
(TIF)

Table S1 List of the primers used in the genotyping of the *C. briggsae* introgression fragments in *C. sp.9*.
(XLSX)

Table S2 List of *C. briggsae* mutants in the two-factor mapping.
(XLSX)
