# Supplementary figures and images for: A Method for Rapid and Simultaneous Mapping of Genetic Loci and Introgression Sizes in Nematode Species
Source: PLoS One. 2012 Aug 31;7(8):e43770. doi: 10.1371/journal.pone.0043770 (PMC3432054; doi:10.1371/journal.pone.0043770)

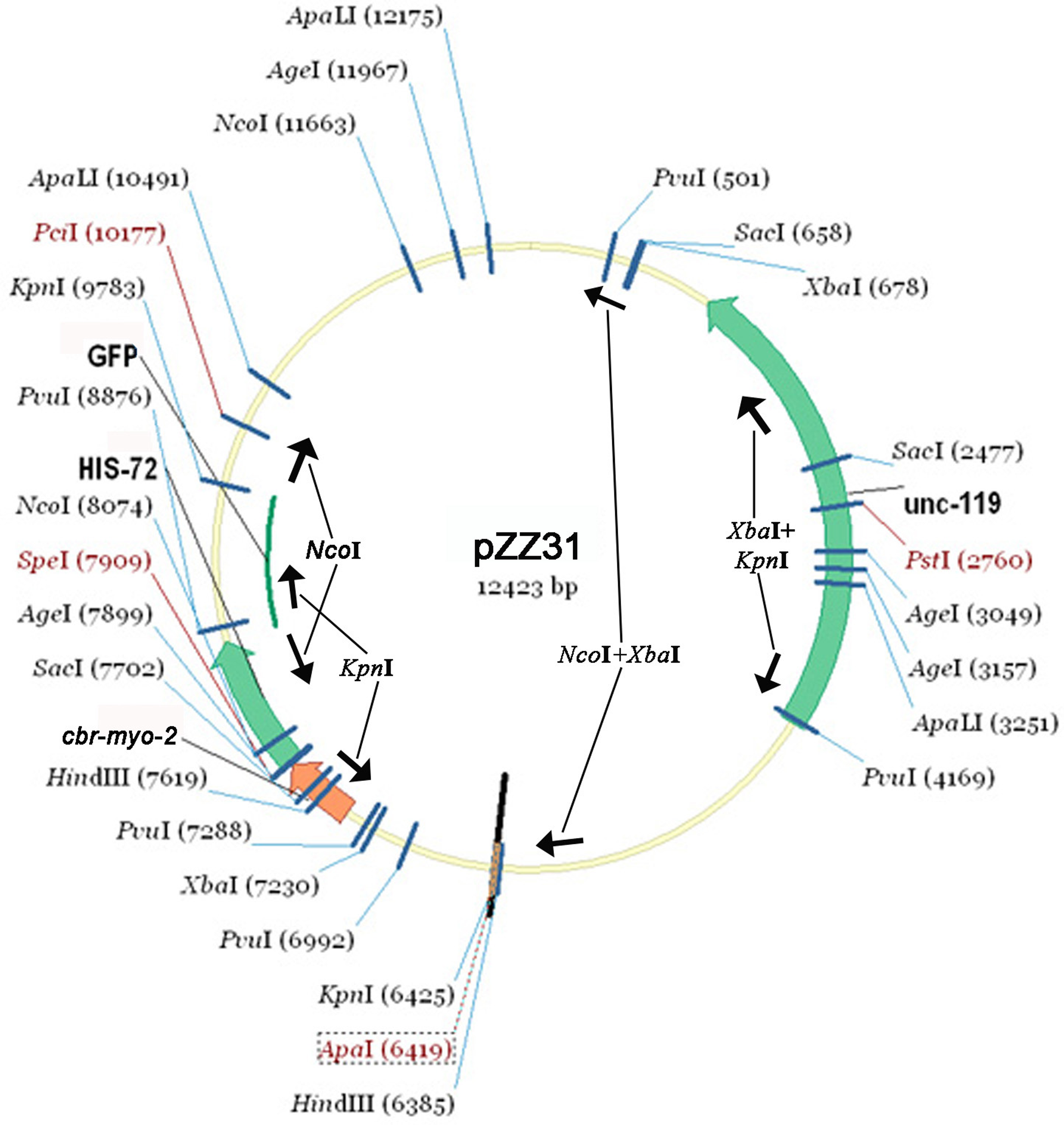

Supplement: Figure S1 — Relative positions of PCR primers and its combinations with restriction enzymes as shown in the context of the restriction map of the construct pZZ31 that was used to bombard the cbr-unc-119 mutant for generation of the stable transgenic line expressing GFP. The ORFs are self-explanatory. (TIF) [file pone.0043770.s001.tif]

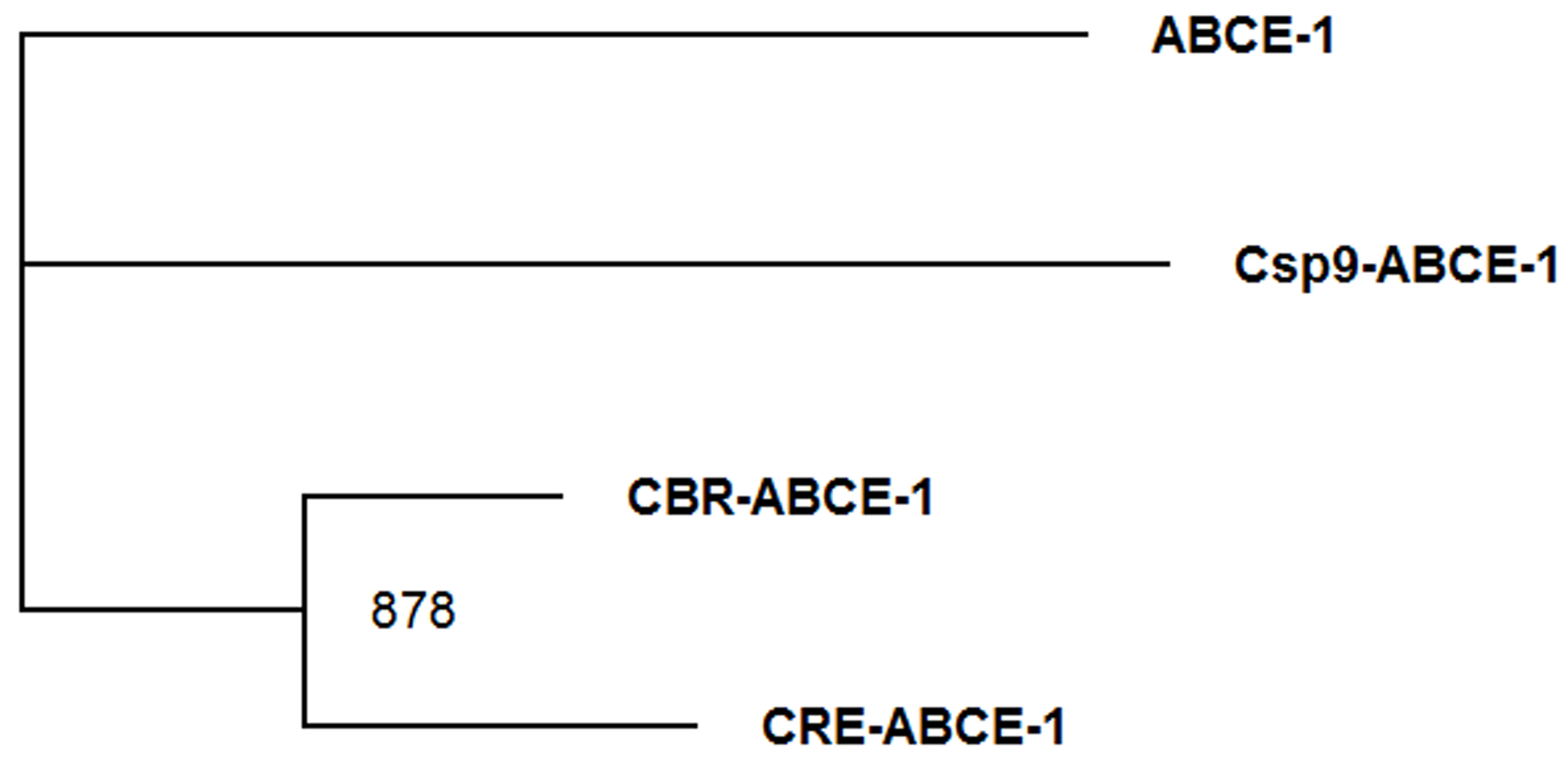

Supplement: Figure S2 — The unrooted Neighbor-Joining (NJ) tree constructed with multiple alignment using ABCE-1 protein sequence from C. elegans (ABCE-1), C. sp.9(Csp9-ABCE-1), C. briggsae (CBR-ABCE-1) and C. brenneri(CRE-ABCE-1). The number of bootstrap support from 1000 replicates was shown. (TIF) [file pone.0043770.s002.tif]
